# Adolescent Health and Parents’ and Teachers’ Beliefs about Smoking: A Cross-Sectional Study

**DOI:** 10.3390/children11091135

**Published:** 2024-09-19

**Authors:** Francisco Carrión-Valero, Joan Antoni Ribera-Osca, José M. Martín-Moreno

**Affiliations:** 1 Service of Pneumology, Hospital Clínico Universitario, Avenida de Blasco Ibáñez 17, El Pla del Real, 46010 Valencia, Spain; 2 Department of Medicine, Universitat de València, 46010 Valencia, Spain; 3 Health Care Center Alcàsser, Department of Health Valencia-La Fe, Alcàsser, 46290 Valencia, Spain; ribera_juaosc@gva.es; 4 Department of Preventive Medicine and Public Health, Universitat de València, 46010 Valencia, Spain; jose.martin-moreno@uv.es

**Keywords:** adolescents, lifestyle, parental smoking, teachers smoking, tobacco, secondary education, survey study

## Abstract

Background: The acquisition of healthy lifestyle habits by adolescents is largely influenced by close interpersonal relationships including their parents and teachers. Methods: A cross-sectional survey regarding tobacco use was conducted among 304 parents and 41 teachers of secondary school adolescents (12–17 years of age) enrolled in the first, second, and third grades of compulsory secondary education at the largest high school in Sueca, Valencia, Spain. Results: The prevalence of smoking was 36.2% among parents (occasional smokers 75.4%) and 19.5% among teachers (occasional smokers 62.5%). Most parents (89.8%) did not believe that their child smoked and 95.7% viewed it negatively if they did. Also, 75.2% of parents agreed that tobacco consumption encourages adolescents to use cannabis or other drugs. Friends who smoke and additives added to cigarettes were considered very influential factors for starting smoking. Most teachers (90.2%) reported having given a recommendation to their students to quit smoking. Cannabis and cigarette smoking were rated as harmful to health. Most respondents believed that cigarette smoking may favor the consumption of cannabis. Conclusions: The use of e-cigarettes was also considered a risk factor for starting smoking. It would be advantageous incorporating parents and teachers as role models in multidisciplinary interventions addressing smoking behavior in secondary school students.

## 1. Introduction

A healthy lifestyle in adolescence is a multidetermined behavior, influenced by overlapping combinations of psychosocial, biological, and environmental factors. The adoption of positive behaviors, such as regular physical exercise, a balanced diet, and avoidance of consumption of harmful substances, has been recognized as an essential factor for healthy adolescent development and health [1]. Additionally, maintaining a healthy lifestyle during adolescence is linked to extended benefits in adulthood with increased psychological well-being contributing to a better quality of life [2].

Parents constitute a fundamental influence on the tobacco consumption of their children, not only through smoking-related specific behaviors, such as allowing smoking at home, being active smokers themselves, or discussing the topic with their children [3,4], but also through the quality of their relationships [5], the affection they show towards their children, and parenting understanding of their problems [6]. On the other hand, it has been shown that teachers serve as important role models and adolescents who have seen school members smoking are more likely to approve smoking as usual or a socially acceptable behavior [6,7].

The family and school environment are two essential determinants that can either facilitate or hinder the initiation of tobacco consumption in adolescents, and there is consistent evidence of the importance of smokers among family members, teachers, and peers as risk factors for adolescents’ smoking behavior [8,9]. Although regarding smoking in schools and access points to schools, Spain has one of the most restrictive smoke-free legislation in Europe [10]; the Survey on Drug Use in Secondary Education in Spain (ESTUDES 2022) of a representative sample of teenagers aged 14–18 years showed that almost 40% of students reported having seen teachers smoking tobacco at their educational centers, 60% have witnessed other students smoking within the school environment, and 39.1% admitted to living in households where other persons smoke daily [11]. High levels of visibility of teachers smoking on school grounds increased the odds of being a smoker among students [12]. The relationship between the smoking status of teachers and adolescent smoking has important implications when designing comprehensive adolescent smoking prevention programs [13].

Assessment of the parents’ and teachers’ attitudes and opinions about smoking can provide important information on their role in influencing the behavior of adolescents about quitting or preventing them from starting to smoke. Therefore, the present cross-sectional survey study was conducted to evaluate the smoking habits, prevalence, and attitudes in a sample of parents and teachers of adolescent students of secondary school in Valencia, Spain.

## 2. Materials and Methods

### 2.1. Design and Participants

A cross-sectional survey study was carried out in a representative sample of parents and teachers of adolescents, aged 12 to 17 years, who attended first, second, and third grade of compulsory secondary education (Educación Secundaria Obligatoria, ESO) at a major high school in the city of Sueca (28,086 inhabitants, 2023 census) in the autonomous community of Valencia, Spain. Sueca is the capital of the region named Ribera Baja and possesses about 4 miles of Mediterranean coastline. The major high school selected for the study is a public center where compulsory secondary education, baccalaureate degree, and professional training cycles are taught. The total number of students is around one thousand. A preventive multi-personal intervention model against tobacco consumption was implemented in a sample of 306 students belonging to 14 classes of the first, second, and third ESO grades; the results of which were reported in a previous publication [14].

For the objectives of the present study, parents and teachers of the adolescent students targeted by the intervention, that is, students 12–17 years enrolled in first, second, and third ESO grades during the 2017–2018 academic year, were invited to participate in a survey study and to complete a study questionnaire regarding different aspects of tobacco smoking and drug use. The primary objective of the study was to collect data on parents’ and teachers’ beliefs about smoking, which could indirectly contribute to gathering information on their potential role as models, which would encourage their adolescent children to develop healthy lifestyle habits.

Parents were recruited taking advantage of the informative meetings with tutors of each ESO class of the first, second, and third grades, which took place at the beginning of the academic year. At these meetings, parents were provided with an informative brochure that included explanations of what parents can do to protect children from tobacco smoke and how they can act as models to encourage non-smoking initiation and support smoking cessation among adolescents who smoke. Parents who voluntarily agreed to take part in the study signed a written informed consent and completed a short and anonymous study questionnaire at the end of the informative session. Teachers of the 14 classes of the corresponding ESO grades were also approached asking for their participation, which involved completing the study questionnaire and providing written informed consent.

The study was approved by the Ethics Committee of the Health Research Institute of the Valencian ‘Hospital Universitari i Politècnic La Fe’ (code 2016/0599, approval date 17 January 2017). All participants signed the written informed consent form.

### 2.2. Study Questionnaire

Parents and teachers completed the same study questionnaire. A 38-item ad hoc study questionnaire was developed to collect demographic data, smoking status (age of onset of consumption and reasons and attempts to quit smoking for those who were current smokers), beliefs about influential factors that lead their children/students to smoke, opinions regarding e-cigarettes, cannabis consumption, and level of communication with their children/students. The study questionnaire used in the parents’ and teachers’ surveys is shown in Table S1 of the Supplementary Material.

### 2.3. Statistical Analysis

Categorical variables are expressed as frequencies and percentages and continuous data as mean and standard deviation (SD). An ordinal logistic regression analysis was performed to assess the relationship between the parent’s smoking condition and the probability of students being smokers. Data are expressed as odds ratio (OR) and 95% confidence interval (CI). Statistical significance was set at *p* < 0.05. Statistical analysis was performed with the R statistical package (version 3.6.1).

## 3. Results

### 3.1. Parents’ Survey

A total of 304 parents participated in the survey, 84 fathers and 220 mothers (72.4%), with a mean (SD) age of 45 (5) years. They were mostly Spanish (94.7%) and 80% were married. Secondary education was reported in 33.5% of participants, with university studies in 8.9%. The main sources through which they received information about tobacco included the internet (websites) followed by media and family members. When asked about the preferred method of receiving this information, websites remained in first place, followed by healthcare professionals and tobacco-related talks or courses.

Most parents (89.8%) did not believe that their child smoked and the majority (95.7%) viewed it as bad if they did.

Regarding the parents’ smoking status (Table 1), 110 were smokers, with a prevalence of 36.2%. The percentage of daily smokers was 26.3% (*n* = 80) and occasional smokers 9.9% (*n* = 30). Never smokers accounted for 36.5% of cases and ex-smokers for 27.3%.

Passive smoking was considered harmful for the people around smokers by 82.5% of participants, and 97.3% of parents would not like their child to smoke. Also, 75.2% of the surveyed parents agreed or strongly agreed that tobacco consumption encourages adolescents to use cannabis or other drugs. When they were asked about their opinions regarding cannabis consumption, 92.5% reported that it was frequent or very frequent, and 86.8% believed that tobacco and cannabis were consumed together. Cannabis was rated as the most harmful to health by 14.8% of parents, tobacco as the most harmful by 11.5%, and both cannabis and tobacco to the same extent by 73.7%.

**Figure 1:**
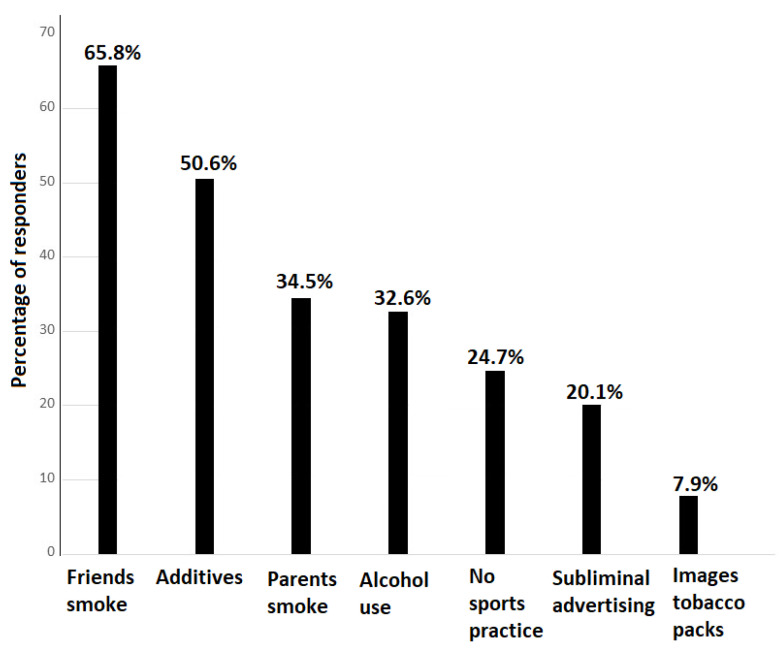
Percentage of parents who rated as “very influential” the different factors that may promote adolescents to start smoking.

Figure 1 shows the percentage of parents who rated different factors as “very influential” for young people starting to smoke. The highest percentages were recorded for the fact that their friends smoke (65.8%), the additives added to tobacco (50.6%), the parents smoke (34.5%), and alcohol consumption (32.6%). Those factors with the highest percentages of responses as “not at all influential” were the images or appearance of tobacco packs (25.3%) and not practicing sports (11.2%). The distribution of responses to each individual factor is shown in Table 2.

In relation to the importance of e-cigarettes, 71% of parents believed that the use of e-cigarettes is harmful to health and 69.7% agreed or strongly agreed on the influence of e-cigarettes to start smoking (Table 3).

Regarding communication with their children, it was considered “very good” by 53.3% of parents, “good” by 44.4%, and “insufficient” by 2.3%. However, only 35.7% recognized to have available an appropriate space to discuss drug consumption with their children.

Finally, the results of logistic regression analysis showed lower probabilities of students being smokers if their parents did not smoke in front of them (OR 0.195, 95% CI 0.053–0.61, *p* = 0.008) or were non-smokers (OR = 0.348, 95% CI 0.169–0.704, *p* = 0.004).

### 3.2. Teachers’ Survey

A total of 41 teachers who taught different materials in the first, second, and third ESO grades participated in the study. There were 10 men and 31 women (75.6%), with a mean age of 45.8 years. Smoking status is shown in Table 4. The prevalence of current smoking was 19.5% (n = 8) and only 7.3% (n = 3) reported smoking on a daily basis. Non-smokers including never smokers and ex-smokers accounted for 80.5% (n = 33) of teachers.

Among teachers who smoked, only 1 of them never made an attempt to quit, 3 had made one attempt, 1 had three attempts, and 3 had more than three attempts. Most teachers (75%) acknowledged that they smoked more or had been doing it for longer than desired, but 87.5% declared that their smoking habit had not made them abandon any usual activity.

None of the teachers smoked at school or in forbidden places and none had tried e-cigarettes, which in turn, 65.8% considered that smoking e-cigarettes were harmful to health. More than half (51.2%) strongly agreed that the consumption of e-cigarettes could be an influence for starting smoking cigarettes and 85.7% agreed or strongly agreed that cigarette smoking may favor the consumption of cannabis. Consumption of cannabis was considered frequent or very frequent among adolescents by 82.9% of teachers.

Health was also the most important reason not to smoke (100% agreement) and 90.2% reported having given a recommendation to their students to quit smoking. Also, 48.8% of teachers believed that advertising was an influential factor for adolescents starting smoking.

Regarding the future, 62.5% believed that they would continue smoking but not on a daily basis, and only 12.5% believed that they would quit next year.

## 4. Discussion

This cross-sectional study provides information on the opinions and attitudes about smoking in a sample of parents and teachers of adolescents aged between 12 and 17 years who were students of first, second, and third ESO grades in a public high school center in Sueca, Valencia, Spain. At the time of the study, these grades included 14 classes with about 25 students per class, so it may be expected that approximately the parents of 350 adolescents would attend the informative sessions at the beginning of each academic course. Therefore, the sample of 304 parents who completed the questionnaire may be considered representative of the target population, but the number of 41 participating teachers was relatively small, probably because they were approached through the tutors of the different classes instead of organizing a joint meeting for their recruitment.

Interestingly, there was a high prevalence rate of smoking of 36.2% among parents surveyed, although occasional smokers accounted for 75.4% of all smokers. This prevalence of 19.5% was notably lower in the teachers’ population, in which 62.5% were also occasional smokers. In a sample of 406 parents of adolescents aged 12–17 years included in the New England Family Study (NEFS), the prevalence of smokers was 26.3% with criteria of nicotine dependence in 14.8% [15]. This study also showed that among adolescents with parents who were nicotine dependent, each previous year of exposure to parental smoking increased the likelihood that adolescents will be in a higher-risk smoking trajectory and progress to regular smoking, supporting the need to address parental smoking cessation as an important strategy to reduce youth smoking risk. However, counseling interventions encouraging parents to quit smoking for their children’s benefit have shown a modest impact on smoking cessation rates [16]. We have also found that the behavior of parents who were non-smokers or did not smoke in front of their children was a significant independent factor in the likelihood of offspring smoking. This finding further confirms the influence of parental smoking on adolescent smoking behaviors consistently reported in the literature [3,8,9,15,16]. It should be noted, however, that the lack of data on actual teen tobacco-use behavior and parental use of other nicotine delivery products is a weakness of the study. Moreover, a potential correlation between teachers’ behavior and the smoking status of students was not evaluated. It would be interesting to repeat a similar survey assessing the correlation between parental and teacher smoking behavior and actual tobacco use by adolescents. Since the majority of smokers start smoking at the adolescent age, the influence of the teachers’ behavior could be crucial for adolescents’ future lifestyle. None of the teachers smoked at school and on all school premises (regulated by a comprehensive Spanish governmental ban) and 90.2% had provided recommendations to their students to quit smoking. However, the prevalence of smokers was 19.5% higher than the 7.8% reported in a study among 495 secondary school teachers in Malaysia [17]. Also, in two French surveys before and following anti-smoking policies, including 2931 teachers in 1999 and 3702 in 2005 aged 20–59 years, the prevalence decreased significantly from 25.7% to 18.2% [18]; this last figure is consistent with our findings. Data reported in 2014 from the Control of Adolescent Smoking (the CAS study) in seven European countries with marked differences in terms of smoke-free schools, ranging from as little as 1% of schools in Denmark to 65% in Norway, clearly showed that restrictive smoking control policies at national and local levels seem to be effective in reducing non-smokers’ exposure to environmental tobacco smoke in school [19].

E-cigarettes are perceived as less harmful than regular cigarettes, but aerosol can contain harmful and potentially harmful substances, including nicotine, heavy metals like lead, volatile organic compounds, and cancer-causing agents [20]. In our study, a high percentage of parents (71%) and teachers also (69.7%) believed that the use of e-cigarettes was harmful to health and agreed on the influence of e-cigarettes to start smoking. Data from the 2023 Annual National Youth Tobacco Survey showed that among U.S. middle and high school students, e-cigarette products were the most used tobacco product (7.7%; 2.13 million) with close to 40% of high school students using e-cigarettes reported frequent use and 29.9% daily use [21]. Availability of flavored products, marketing, and misperceptions regarding harm continue to influence tobacco product use, particularly e-cigarettes, among adolescent students.

Parents also recognized the influence of friends as a highly influential factor for adolescent smoking. Peers and the popularity of peers have been identified as drivers of smoking adoption among youth and the desire for social status as an important motivation [22]. Teachers and parents reported that cigarette smoking may favor the consumption of cannabis. A growing literature has documented the substantial prevalence of and putative mechanisms underlying co-occurring cannabis and tobacco use. In a systematic review of 28 studies, cannabis users who also smoke tobacco were more dependent on cannabis and had more psychosocial problems and poorer cessation outcomes than those who use cannabis but not tobacco [23]. In the present survey, more than 85% of parents believed that tobacco and cannabis were consumed together, but both cannabis and tobacco were considered harmful to health to the same extent by more than 70% of parents. A high percentage of teachers also believed that tobacco is a risk factor for e-cigarette consumption. Co-users and exclusive cigarette smokers have demonstrated comparable levels of biomarkers of exposure to harmful chemicals and toxicants [24]. Therefore, it is critical to consider how concurrent cannabis use may influence health-related outcomes among adolescent smokers [25]. Co-use of e-cigarettes and cannabis is common among youth. Indeed, using tobacco and cannabis together has been associated with greater nicotine addiction, making it more difficult to quit. In a survey of high school students carried out in California in 2020/2021, co-use of tobacco and cannabis was most likely associated with absenteeism and lower grades, so educational outcomes could be improved by comprehensive efforts to prevent or reduce youth substance co-use [26].

More than half of parents reported good relationships with their offspring. The influence of the family environment has been identified as an important etiology for adolescent smoking, and family-based programs may be considered when targeting adolescent anti-smoking interventions. Family dynamics characterized by openness and clarity of communication may have a preventive function for adolescent smoking behavior [27].

The present findings should be interpreted considering the limitations of the study, including a convenience sample of parents and teachers selected from a single high school. Expanding the sample size with more schools and regions would contribute to increasing the representativeness and generalizability of the findings. As the number of participating teachers was relatively small, organizing joint meetings for recruitment to increase participation would be necessary in the design of future studies. Also, the fact that differences in responses between smoking and non-smoking parents and teachers were not evaluated nor between male and female participants should be considered a limitation of the present study. However, strengths of the study include the large size of the parents’ sample as well as there is relatively little information based on cross-sectional survey studies on the perspectives of parents and teachers regarding different aspects related to smoking in adolescents.

## 5. Conclusions

This study identifies some characteristics of parents and teachers associated with their beliefs and opinions regarding tobacco use in secondary school adolescents. The prevalence of daily smokers was 26.3% among parents and 19.5% among teachers. Most parents did not believe that their children smoke and if they did, they would consider it bad. Influential factors related to starting smoking included the fact that friends smoke, additives added to cigarettes, and the use of e-cigarettes. Most teachers recognized having given a recommendation to their students to quit smoking. Cannabis and cigarette smoking were rated as both harmful to health, and the majority of respondents believed that cigarette smoking might favor the consumption of cannabis. The present findings suggest the convenience of incorporating parents and teachers as role models in multidisciplinary interventions addressing smoking behavior in secondary school adolescents. Further studies with longitudinal design to track changes in attitudes and behaviors over time would be helpful for assessing interventions to quit smoking involving adolescent students, parents, and teachers.

## Figures and Tables

**Table 1:** Smoking status in the parents’ population.

Smoking Status	Number (%)
All parents	304 (100)
Never smoker	111 (36.5)
Ex-smoker	83 (27.3)
Current smoker	110 (36.2)
Smoker on a daily basis	80 (26.3)
Occasional smoker	30 (9.9)

**Table 2:** Parents’ opinions on the level of influence of different factors regarding adolescents’ behavior to start smoking.

Factors	Very Influential n (%)	Somewhat Influential n (%)	Slightly Influential n (%)	Not at All Influential n (%)
Friends smoke	200 (65.8)	74 (24.3)	28 (9.2)	2 (0.6)
Additives added to tobacco	154 (50.6)	91 (29.9)	33 (10.8)	26 (8.5)
Parents smoke	105 (34.5)	115 (37.8)	59 (19.4)	25 (8.2)
Alcohol consumption	99 (32.6)	152 (50)	42 (13.8)	11 (3.6)
Do not practice sports	75 (24.7)	110 (36.2)	85 (28.0)	34 (11.2)
Subliminal advertising	61 (20.1)	124 (40.8)	94 (30.9)	25 (8.2)
Images/appearance of tobacco packs	24 (7.9)	76 (25)	127 (41.8)	77 (25.3)

**Table 3:** Parents’ opinions regarding e-cigarettes.

Data	Number (%)
Total parents	304 (100)
Do you think that e-cigarettes are harmful to health?	
Yes	216 (71.0)
No	23 (7.6)
Unknown/no answer	65 (21.4)
Do you think that e-cigarettes can influence to start smoking?	
Strongly agree	86 (28.3)
Agree	126 (41.4)
Disagree	11 (3.6)
Strongly disagree	22 (7.2)
Unknown/no answer	59 (19.4)

**Table 4:** Smoking status in the teachers’ population.

Smoking Status	Number (%)
All teachers	41 (100)
Never smoker	19 (46.3)
Ex-smoker	14 (34.1)
Current smoker	8 (19.5)
Smoker on a daily basis	3 (7.3)
Occasional smoker	5 (12.2)

## Data Availability

The study data are available from one of the authors (J.A.R.-O.) upon request.

## Supplementary Materials

The following supporting information can be downloaded at https://www.mdpi.com/article/10.3390/children11091135/s1, Table S1: Questionnaire for parents and teachers.
