# Supplementary material for: Adolescent Health and Parents’ and Teachers’ Beliefs about Smoking: A Cross-Sectional Study
Source: Children (Basel). 2024 Sep 19;11(9):1135. doi: 10.3390/children11091135 (PMC11430281; doi:10.3390/children11091135)
Supplement: Supplementary file 1 [file children-11-01135-s001.zip › children-3186253-supplementary.pdf]

Supplementary Material

Table S1. Questionnaire for parents and teachers

1. **Sex**:
   - Man
   - Women

2. **Age:** ..... years

3. **Civil status:**
   - Single
   - Married
   - Separated
   - Divorced
   - Widower
   - Other

4. **Who do you currently live with?** (multiple choice)**:**
   - Alone
   - Spouse
   - Partner
   - Father
   - Mother
   - Offspring
   - Brothers
   - Grandparents
   - Other

5. **What is the highest level of education you have completed or passed?:**
   - No studies
   - Primary education
   - Secondary education
   - University studies
   - Other
6. **Have you born in Spain?:**
   - Yes
   - No
   -

7. **What country were you born in?:**

8. **How many years have you been living in Spain?:**

9. **What were the main sources through which you received information about tobacco?:**
   - Internet
   - Social networks
   - Parents
   - Family members
   - Friends
   - Coworker

– Healthcare professionals
– Courses
– Professionals
– Official bodies
– Books and/or leaflets
– Mass media
– Police
– Other

10. What are the main sources through which you would like to receive better and more objective information about tobacco consumption, and tobacco-associated effects and problems and forms of consumption?:

– Internet
– Social networks
– Parents
– Family members
– Friends
– Coworker
– Healthcare professionals
– Courses
– Professionals
– Official bodies
– Books and/or leaflets
– Mass media
– Police
– Other

11. In your opinion, do you think that the fact that the father or mother smokes can influence the child to do so?:

– Strongly disagree
– Disagree
– Agree
– Strongly agree

12. How much do you think the following factors influence young people starting to smoke? (very influential, somewhat influential, slightly influential, not at all influential):

– The parents smoke
– The friends smoke
– Do not practice sport
– The images or appearance of tobacco packs
– Alcohol consumption
– The additives added to tobacco
– Subliminal advertising

13. Do you know or believe that your child/student smokes?:

– Yes, I know
– I don’t think he/she smokes
– I don’t know

14. **If your child/students smokes, what do you think?:**
    - I see it well
    - I see it wrong
    - I don’t care

15. **You are currently:**
    - Non-smoker
    - Occasional smoker
    - Regular smoker
    - Ex-smoker (how long have you been without smoking: days … months… years…)

16. **At what age did you start smoking?:** … years

17. **How many cigarettes do you smoke a day?:**

18. **If you are an occasional smoker:** Number of cigarettes per week …

19. **If you have ever tried to quit smoking:** number of previous attempts …

20. **Why did you stop smoking?:**
    - Starting of having unpleasant symptoms
    - Advice from your family environment
    - Advice from healthcare professional
    - To save money
    - To protect your health
    - To set a good example
    - To not disturb your surroundings

21. **Do you think that smoking is harmful for your health?:**
    - Yes
    - No

22. **Do you think that smoking is harmful to the people around you?:**
    - Yes
    - No

23. **Has a healthcare professional advised you to quit smoking or have you ever received help from a professional to quit smoking?:** Yes (from whom)
    - Family physician
    - Healthcare center nurse
    - Hospital physician
    - Pharmacist
    - Odontologist
    - Other healthcare professional …

24. **Mark those statements with which you agree:**
    - The doctor must convince people to stop smoking
    - The smoker can give up cigarettes willingly
    - It is unpleasant to be around a person who is smoking

- Doctors should lead by example and should not smoke
- You are aware of the risks of cigarettes
- Most smokers would not quit cigarettes even if their doctors advised them to

25. Regarding cigarette advertisements, please select those statement with which you agree:
- Warnings about dangers of cigarettes should be on packages
- Pro-cigarette advertising should be forbidden
- People who wish to smoke should do so in places prepared from them
- The price of cigarettes should be considerably increased

26. Would you like your child/student to smoke?
- Yes
- No
- Tt's his/her decision
- I am indifferent

27. Do you think that electronic cigarettes are harmful to health?:
- Yes
- No
- Unknown/no answer

28. Do you think that electronic cigarettes can influence young people to start smoking?:
- Strongly disagree
- Disagree
- Agree
- Strongly agree

29. Do you think that tobacco consumption encourages young people to consume cannabis or other drugs?:
- Strongly agree
- Agree
- I doubt
- Disagree
- Strongly disagree

30. Do you believe that the use of cannabis (marijuana, hashish) among young people is:
- Uncommon
- Frequent
- Very frequent

31. Do you think that among adolescents the consumption of tobacco and cannabis at the same time is:
- Uncommon
- Frequent
- Very frequent

32. Which of the two products do you consider that is more harmful to health?:
- Cannabis
- Tobacco
- Both to the same extent
- None of them

33. **Do you think that tobacco consumption in the car should be regulated?:**
    - Yes
    - No
    - Only when there are children inside

34. **What do you think are risk factors for tobacco use for your child?:**
    - Accessibility to consumption
    - Influence of the groups of friends
    - Lack of information
    - Other (please specify)

35. **If you child/student smoke, what do you think he/she does it?:**
    - For friends
    - Because smoking at home
    - For personal reasons
    - Because he/she tried it and liked it
    - Other (please specify)

36. **Which do you think is the most addictive substance?:**
    - Tobacco
    - Marijuana
    - Cocaine
    - Alcohol

37. **Do you consider than communication with your child/student is?:**
    - Good
    - Very good
    - Insufficient

38. **Do you have a meeting place with your child/student to discuss topics such as problematic drug use?:**
    - Never
    - Sometimes
    - Always

QUESTIONNAIRE FOR TEACHERS

Sex:
- Man
- Women

Age: ..... years

ESO grade:
- First
- Second
- Third
- Fourth

1. **Your current smoking status is:**
   - Daily smoker
   - Occasional smoker, some days or during the weekend
   - Ex-smoker
   - Never smoker

2. **If you smoke or have smoked, how old were you the first time you tried tobacco (even if it was just a puff)? ______________ years**

3. **Have you ever given advice or recommendations to your students to prevent or discourage tobacco use?**
   - Yes
   - No

4. **Do you think that tobacco advertising in the media (television, radio, cinemas, street signs, nightclubs, bars) influences people to start smoking?**
   - Yes
   - No
   - I don't know

5. **What reason do you think is most important for not smoking?**
   - My health
   - Fashion
   - Saving
   - Do not disturbed
   - Other

Knowledge about electronic cigarettes

6. **In relation to electronic cigarettes you are:**
   - Non-consumer
   - Ex-consumer
   - Occasional consumer
   - Regular consumer

7. **Do you think that the electronic cigarette is harmful to health?**
   - Yes
   - No
   - Unknown/no answer

8. **Do you think that electronic cigarettes can influence young people to start smoking?**
   – Strongly disagree
   – Disagree
   – Strongly agree

9. **Do you think that tobacco consumption encourages young people to consume cannabis or other drugs?**
   – Strongly agree
   – Agree
   – I doubt
   – Disagree
   – Strongly disagree

10. **Do you believe that the use of cannabis (marijuana, hashish) among young people is:**
   – Uncommon
   – Frequent
   – Very frequent

Answer only current smokers

11. **Your current tobacco consumption is:**
   – Occasional (less than once a week)
   – Weekly (I smoke at least once a week but not daily)
   – All days from 1 to 10 cigarettes
   – All days from 11 to 20 cigarettes
   – All days from 21 to 30 cigarettes
   – All days more than 30 cigarettes

12. **When did you smoke the first cigarette?**
   – Before 10 years of age
   – From 10 to 12 years
   – From 13 to 15 years
   – From 16 to 18 years

13. **When did you start to smoke daily?**
   – Not yet
   – Before 10 years of age
   – From 10 to 12 years
   – From 13 to 15 years
   – From 16 to 18 years

14. **Which were the reasons for starting smoking?** (multiple choice)
   – Influence to imitate my best friends
   – Try to imitate my idols (soccer, music, …)
   – To imitate elders (parents and/or relatives, teachers...)
   – Desire to establish relationships or social acceptance with other people
   – Due to the influence of tobacco advertising
   – Out of curiosity (to know what it feels like to smoke)
   – Because they prohibited me
   – Other reasons (write): __________

15. **If you decide to quit smoking, what do you think will be the main reasons** (multiple choice)
    - I have no specific reasons
    - Do not contract possible diseases
    - Own decision due to health problems
    - Family/social prohibition/recommendation
    - Respect to non-smokers
    - High price of tobacco
    - My doctor recommends it to me
    - Other: ____________________________________

16. **What has been the longest time that you have been without smoking?**
    - I haven't left smoking
    - From 24 to 48 hours
    - From 3 to 7 days
    - More than 1 week
    - From 7 to 30 days

17. **Do you smoke in places in which smoking is not allowed within the high school?**
    - Yes
    - Occasionally
    - Never

18. **Answer YES or NO to the following questions:**
    - Do you think you smoke more or have been smoking for longer, what you originally wanted?
    - Have you tried to quit smoking or would like to quit smoking?
    - Did you have reduced or stopped sports or another activity due to tobacco consumption?

19. **How long does it take from when you wake up until you smoke your first cigarette?**
    - Less than 5 minutes
    - From 6 to 30 minutes
    - From 31 to 60 minutes
    - More than 60 minutes (write the time):

20. **When do you smoke more often in the morning or afternoon?**
    - In the morning
    - In the afternoon

21. **Which cigarette gives you the most satisfaction or would be more difficult for you to quit?**
    - The first of the day
    - The one after meals
    - Any other

22. **Do you smoke when you are sick?**
    - Yes
    - No

23. Do you smoke in forbidden places (movies, bus, school, hospitals, …)?

- Yes
- No

24. How do you foresee your behaviour in the coming years?

- I will continue smoking daily
- I will not smoke daily
- I will quit
